# RUSHing to the Diagnosis: Aortic Abdominal Aneurysm Detected Using the Rapid Ultrasound for Shock and Hypotension (RUSH) Protocol in the Wards

**DOI:** 10.7759/cureus.32565

**Published:** 2022-12-15

**Authors:** Jose A Rivera, Daniel Aragon, Percy Thomas, Paul Dominici, Michael Menowsky, Olabiyi O Akala

**Affiliations:** 1 Internal Medicine, Doctors Hospital at Renaissance/University of Texas Rio Grande Valley, Edinburg, USA; 2 Emergency Medicine, Doctors Hospital at Renaissance/University of Texas Rio Grande Valley, Edinburg, USA

**Keywords:** rush, internal medicine, leaking aortic abdominal aneurysm, ultra sound, pocus

## Abstract

The rapid ultrasound for shock and hypotension (RUSH) protocol is a useful tool used in the emergency department (ED) when addressing the severity and etiology of shock. It was designed to be performed in under two minutes with evaluation of the pump (heart), tank (inferior vena cava, thoracic and abdominal compartments) and the pipes (large arteries and veins). However, its application or one similar should extend beyond the ED and into the hospital floor. Here we present an 80-year-old gentleman with a history of atrial fibrillation (A-Fib) on anticoagulation who arrived at the ED due to an episode of pre-syncope just prior to arrival. Initial EKG is concerning for A-Fib with rapid ventricular response (RVR) with a rate in the 130s. After fluid resuscitation patient improved and he was admitted to the telemetry floor for further cardiac workup and cardiology consultation. While waiting for a room in the ED, patient became hypotensive, diaphoretic and pale. After complaining of lower abdominal pain, the ED physician performed a RUSH which showed an abdominal aorta of 8 cm concerning for dissection. Diagnosis was confirmed with CT angiography of the abdomen and he was taken to the OR with successful repair of the abdominal aortic aneurysm (AAA). Patient made meaningful recovery and was discharged to in-patient rehab.

The patient described in this vignette was delayed in the ED due to lack of beds on the floor. This allowed for quick ultrasound work-up by the ED physician which led to immediate recognition of the AAA and immediate response by the vascular surgery team. Should this patient have been on the hospital floor, it is unclear if such prompt steps would have occurred prior to patient’s further hemodynamic demise.

## Introduction

The rapid ultrasound for shock and hypotension (RUSH) protocol is a useful tool used in the emergency department (ED) when addressing the severity and etiology of shock [1-3]. Such shock states include hypovolemic, cardiogenic, obstructive and distributive. It is designed to be performed in under two minutes with evaluation of the pump (heart), tank (thoracic and abdominal compartments) and the pipes (large arteries and veins) [3]. However, its application or one similar should extend beyond the ED and into the hospital floor. Studies have revealed that hospital rapid response teams equipped with a protocolized use of a handheld point-of-care ultrasound (POCUS) device reached a diagnosis quicker, time to treatment/intervention is shorter and in-hospital mortality rates are lower [1-2]. In this case report, we aim to encourage the use of ultrasonography, specifically a POCUS, on the undifferentiated shocked patient not only in the ED or intensive care unit (ICU) but also in the wards. 

## Case presentation

Patient is an 80-year-old gentleman with a past medical history of atrial fibrillation on rivaroxaban that arrives to the ED with a complaint of dizziness and pre-syncope that began just prior to arrival. Emergency medical services (EMS) reports blood pressure (BP) in the field of 60s/40s and one liter of normal saline is started.

Patient is evaluated by the ED physician and further complains of nausea, vomiting and diaphoresis. Initial EKG is concerning for atrial fibrillation with rapid ventricular response (A-Fib with RVR) which resolved after the one liter of normal saline started by EMS. Vitals throughout admission and labs on presentation are available in Tables 1, 2. At approximately 19:40 patient is rate controlled, blood pressure improves, labs are unremarkable and he is admitted to the telemetry floor for further cardiac workup. Cardiology is consulted by the ED physician and patient is placed on digoxin due to borderline hypotension.

**Table 1:** Vital signs while the patient was located in the Emergency Department. HR: heart rate, BP: blood pressure, MAP: mean arterial pressure

Time	HR	BP	MAP
18:58 (Time of arrival)	102	84/54	66
19:40	91	107/56	73
22:05	93	95/64	74
01:13	98	96/71	79
04:42 (Episode of shock)	38	76/51	59
04:47	110	78/31	47
05:15	113	86/41	56
05:37	87	86/40	49

**Table 2:** Labs on admission Leukocytosis with no anemia noted at the time of admission. WBC: white blood cells, Hgb: hemoglobin, Hct: hematocrit, Plt: platelet

Test	Result
WBC	16.9 (4.8-10.9 th/uL)
Hgb	11.0 (10.8-14.7 g/dL)
Hct	35.40% (32.2%- 42.9%)
Plt	263 (146-388 th/uL)
Troponin	0.02 (0.00-0.04 ng/mL)

At approximately 4:42 am, while holding in the ED for a telemetry room, patient became briefly bradycardic, hypotensive (Table 1) with associated diaphoresis, pallor and new onset lower abdominal pain. Labs in the ED are obtained again via i-STAT (Table 3) and RUSH is performed by ED physician which revealed an abdominal aorta of approximately 8 cm concerning for dissection. 

**Table 3:** Labs at the time of undifferentiated shock Labs obtained at the time of undifferentiated shock which resulted approximately one hour after presentation with significant drop in H/H. Hgb: hemoglobin, Hct: hematocrit

Test	Results
Hgb	7.5 gm/dL (10.8-14.7 gm/dL)
Hct	22% (32.2%- 42.9%)

**Figure 1:**
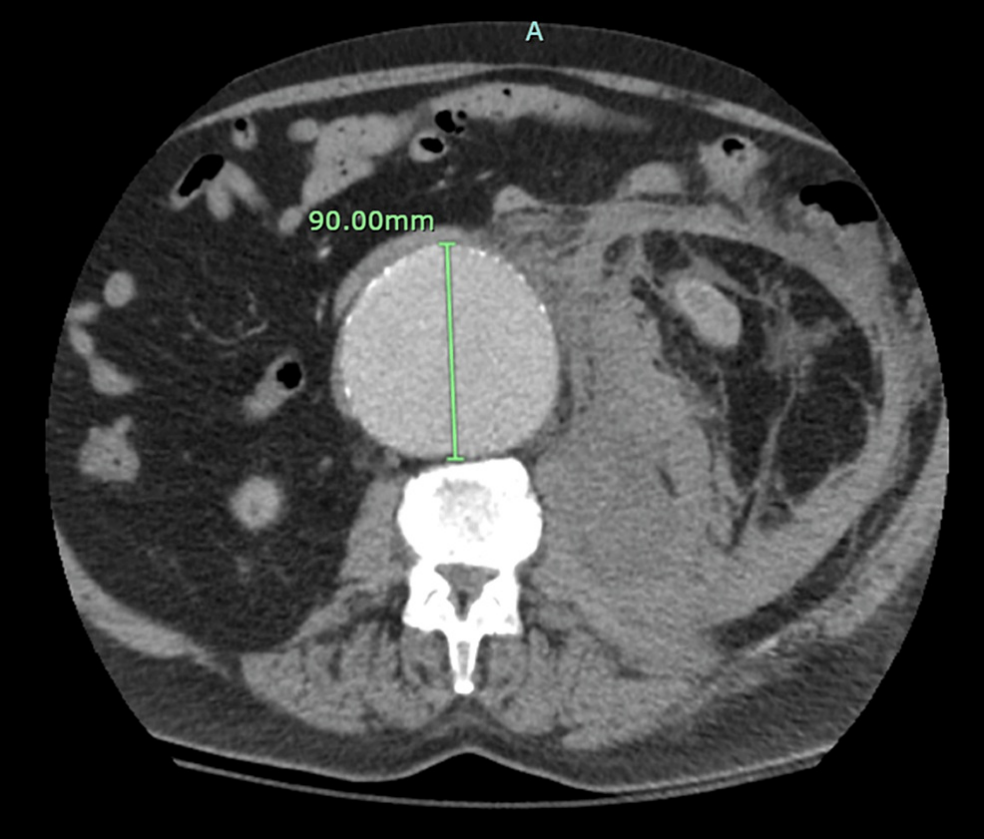
CT angiography abdomen transverse view Large infrarenal aortic aneurysm with an acute leak just below the origin of the left renal artery with a large amount of acute left retroperitoneal hemorrhage.

**Figure 2:**
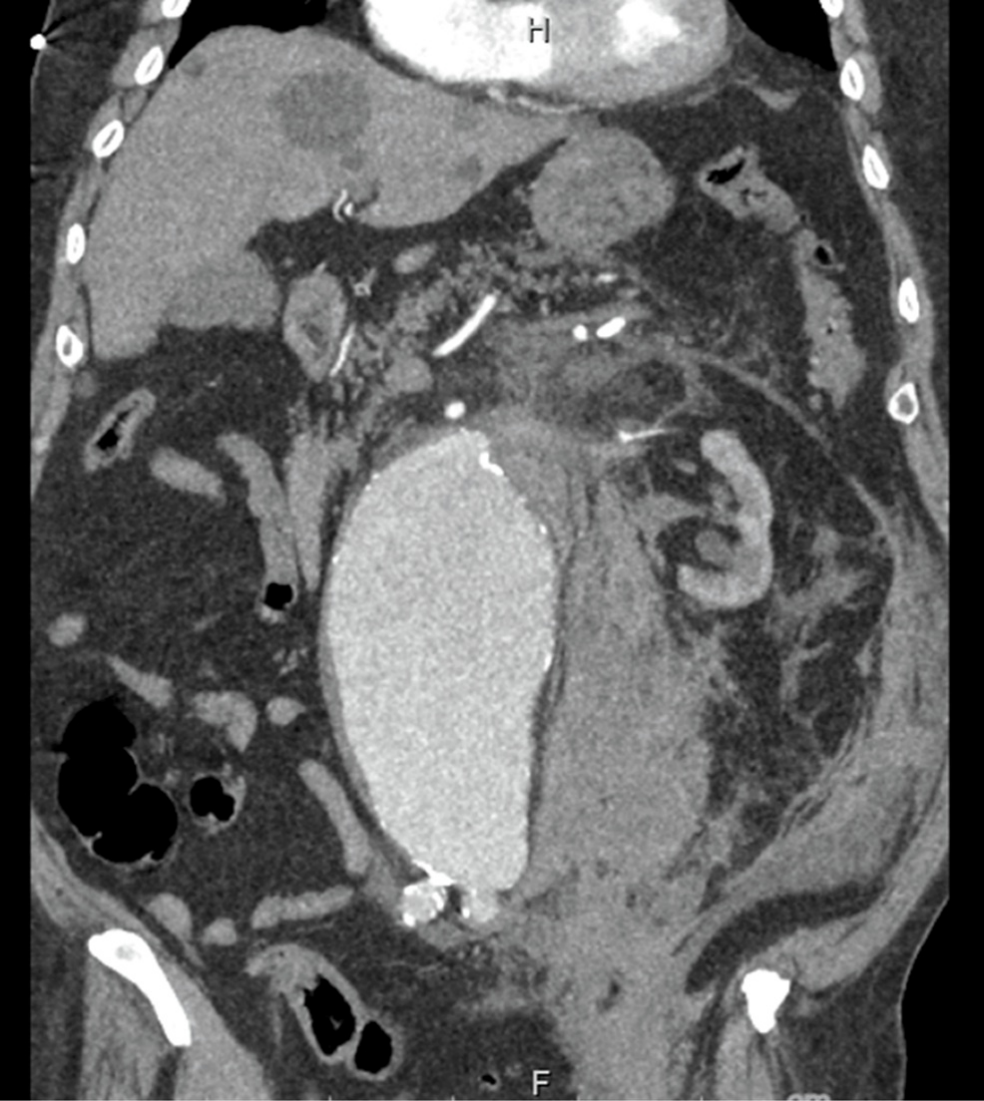
CT angiography abdomen coronal view Large infrarenal aortic aneurysm (blue arrow) with an acute leak just below the origin of the left renal artery with a large amount of acute left retroperitoneal hemorrhage (red arrow).

**Figure 3:**
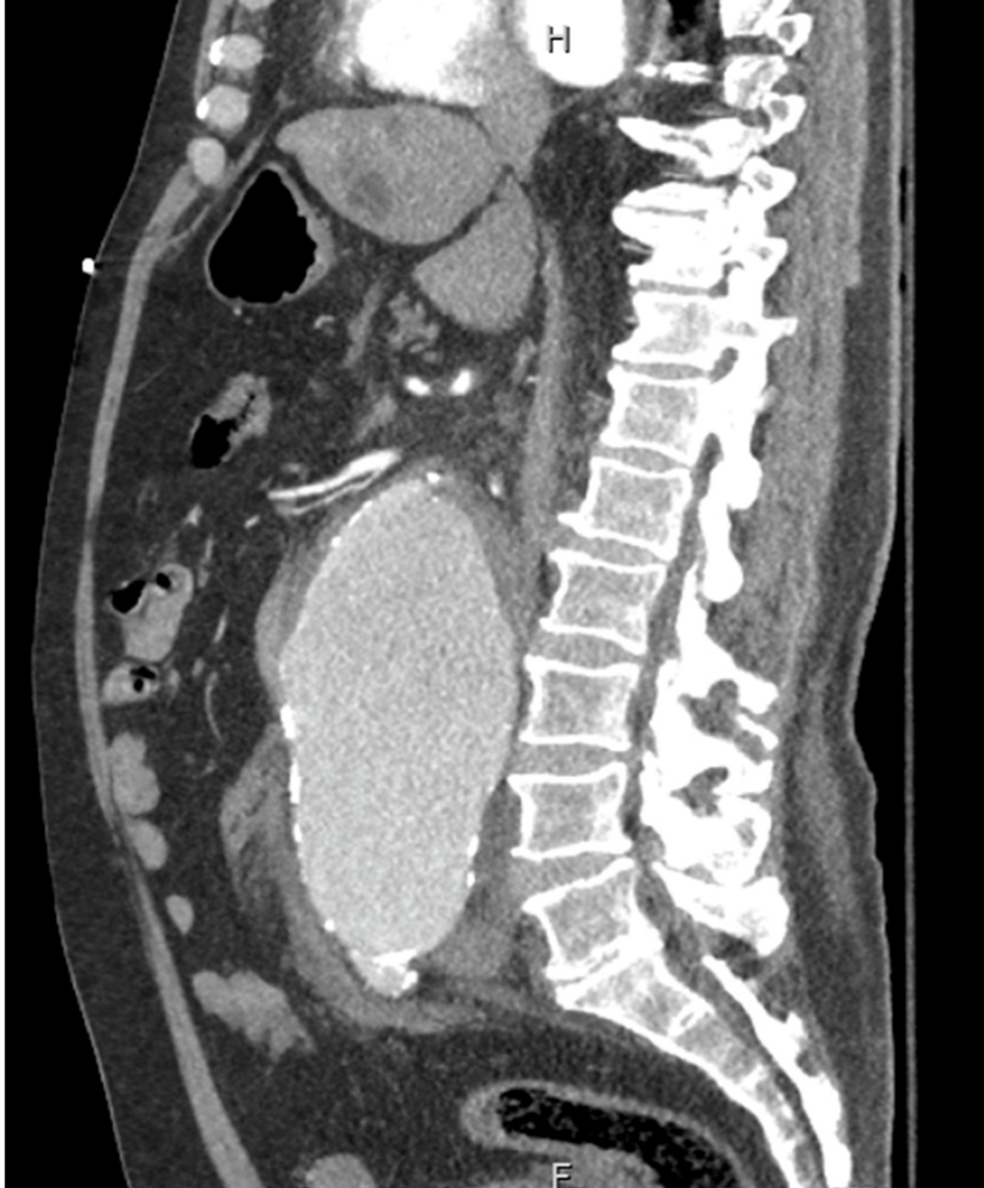
CT angiography abdomen sagittal view Large infrarenal aortic aneurysm with an acute leak just below the origin of the left renal artery with a large amount of acute left retroperitoneal hemorrhage (red arrows).

CT angiography of the abdomen is performed approximately 13 minutes after POCUS which confirms an aortic abdominal aneurysm (AAA) measuring 9.5 x 8.7 cm in cross-section with significant amount of extravasation into the left retroperitoneum (Figures 1-3). Reversal of rivaroxaban is done with prothrombin complex concentrate and rapid transfusion of 2 units of packed red blood cells and fresh frozen plasma are administered. Vascular surgery was immediately consulted who successfully repaired the AAA with bifurcated graft to common iliac. Patient recovered and was discharged to in-patient rehabilitation. 

## Discussion

The use of POCUS is of utmost importance when dealing with a patient with undifferentiated shock and its use must expand beyond the ED and or ICU [1-3]. Emergency Medicine residencies and Critical Care fellowships have ultrasound training incorporated within their curriculum, and though many Internal Medicine residencies do incorporate POCUS training in their curriculum, many do not despite the American College of Physicians (ACP) acknowledgment of its importance [4]. The American Medical Association (AMA) affirms that ultrasound imaging is within the scope of practice of appropriately trained physicians [5] and the Society of Hospital Medicine (SHM) encourages hospitalists to use POCUS as it adds value to their bedside evaluation [6-7]. However, the question remains how do we get this training out there? Various facilities have attempted different methods with success in order to add POCUS to their Internal Medicine training from starting a curriculum from scratch with help of the ICU department [4] to having sonography students teach residents [8].

Most hospitals already have in-place rapid response teams equipped with nursing, respiratory therapist and quick access to EKGs, labs and x-rays [9-10]. Including ultrasound availability along with these rapid response codes would be of high benefit to patient prognosis and lead to a decrease in morbidity and mortality [1-2]. In one single-center, prospective, observational, controlled study, the POCUS-trained group which responded to rapid responses in the hospital had an immediate diagnosis 94% of the time in comparison to the 80% in the controlled (non-POCUS trained) group (p = 0.009). Time to first treatment/intervention was shorter in the POCUS group (15 (10-25) min vs. 34 (15-40) min, p < 0.001) and in-hospital mortality rates were 17% in the POCUS group and 35% in the control group (p = 0.007) [1].

Though we highlight the RUSH protocol in this case, there are other protocolized uses of ultrasound such as Bedside Lung Ultrasound in Emergency (BLUE) [11] or Cardiac Limited Ultrasound Exam (CLUE) [12] that can be utilized for fast decision-making in the wards. The RUSH protocol is a comprehensive approach that examines the pump, tank and the pipes with a high clinical accuracy for differentiation of shock [13,14]. It is not limited to an organ system such as BLUE or CLUE and it is more extensive than the Focused Assessment with Sonography for Trauma (FAST). In RUSH, the "cardiac pump" inspects for mechanical stresses, the cardiac systolic function and cardiac output. The "tank" allows for visualization of the inferior vena cava, jugular venous vein status and collection of fluid in the abdomen. The "pipe" section further examines the abdominal arteries and the aorta in order to rule out aneurysms as well as the lower extremities for deep vein thrombosis (DVT) [3,14]. It is important to remember we are no longer limited to inspection, palpation, percussion and auscultation. Thanks to modern medicine, we now have the fifth pillar, insonation [15].

## Conclusions

The patient described in this vignette was delayed in the ED due to lack of beds on the floor. This allowed for quick ultrasound work-up at the specific time of the patient's episode of shock which led to immediate recognition of the AAA and immediate response by the vascular surgery team. Throughout the last couple of years there has been an increased movement to teach more internal medicine residents and medical students the skills to obtain proper POCUS images. However its use still varies from physician to physician or hospital to hospital. Should this patient have been on the hospital floor, where ultrasound is not usually readily available, it is unclear if such prompt steps would have occurred prior to the patient’s further hemodynamic demise. 
